# Case Report: Successful transplantation of a living donor kidney with five renal arteries procured via laparoscopy and back-table vascular reconstruction using the recipient's internal iliac artery

**DOI:** 10.3389/fmed.2025.1553478

**Published:** 2025-07-25

**Authors:** Chenzhen Yu, Limin Zhang, Chunhui Lv, Sheng Chang, Gang Chen, Zhishui Chen, Weijie Zhang, Daqiang Zhao

**Affiliations:** ^1^Institute of Organ Transplantation, Tongji Hospital, Tongji Medical College, Huazhong University of Science and Technology, Wuhan, China; ^2^Key Laboratory of Organ Transplantation, Ministry of Education, Chinese Academy of Medical Sciences, Wuhan, China; ^3^NHC Key Laboratory of Organ Transplantation, Wuhan, China

**Keywords:** multiple renal arteries, laparoscopic living donor nephrectomy, kidney transplantation, vascular reconstruction, case report

## Abstract

**Background:**

Laparoscopic living donor nephrectomy (LLDN) is the preferred technique for living donor kidney transplantation, but multiple renal arteries pose challenges due to increased surgical complexity. While cases with up to seven renal arteries have been reported, the occurrence of kidneys with more than three renal arteries is extremely rare. This report presents a successful retroperitoneoscopic nephrectomy in a living donor with five renal arteries, a case not previously detailed in the literature.

**Case report:**

A 65-year-old female donor with five left renal arteries underwent retroperitoneoscopic LLDN. The kidney was reconstructed *ex vivo* using the recipient's internal iliac artery trunk and its branches. Vascular reconstruction was achieved by anastomosing the donor's renal arteries to the recipient's iliac artery branches, forming a single arterial ostium, which was then anastomosed to the recipient's external iliac artery. The recipient, a 33-year-old male, also underwent concurrent repair of a left inguinal hernia. Postoperative outcomes were excellent, with immediate graft function, no dialysis requirement, and stable renal function at 60 days post-transplant.

**Conclusion:**

This case demonstrates that retroperitoneoscopic LLDN for kidneys with five renal arteries is technically feasible and safe. It highlights the utility of the recipient's internal iliac artery for *ex vivo* reconstruction, expanding the potential for successful transplantation in complex anatomical scenarios.

## Introduction

Laparoscopic donor nephrectomy is the preferred technique for living donor kidney transplantation (1). Multiple renal arteries (MRA) are a risk factor for increased complications following laparoscopic living donor nephrectomy (LLDN) (2).

Anatomically, without considering transplantation, kidneys with up to seven renal arteries have been reported (3, 4). However, the occurrence of kidneys having more than three renal arteries is extremely rare. In 2019, Recto et al. (5) analyzed a series of 20,782 kidneys and found that only 0.24% had more than three renal arteries, while the prevalence of kidneys with five and six renal arteries are even lower, at only 0.02% and 0.01%, respectively. Earlier, in 1928, Adachi et al. studied a series of 1,838 kidneys and reported a slightly higher prevalence of 0.49% for kidneys with more than three renal arteries (4). In the context of living donor kidney transplantation, the incidence of MRA is ~ 21%, typically involving two arteries and occasionally three (6, 7). For instance, Tabbara et al. (8) analyzed 73 living donor kidneys with MRA and found that 84.9% had two renal arteries, 15.1% had three, and none had more than three renal arteries.

Therefore, although many studies suggest that MRA do not constitute a barrier to LLDN (9–11), research on the feasibility of LLDN in cases with more than three renal arteries remains limited. In this case, we successfully performed a retroperitoneoscopic nephrectomy on a living donor with five renal arteries. Each renal artery was reconstructed with the recipient's internal iliac artery, and both donor and recipient achieved smooth postoperative recovery.

## Case report

A 33-year-old male patient was admitted for living donor kidney transplantation after undergoing hemodialysis for over a year. At admission, the patient had a functioning internal jugular vein catheter for hemodialysis access, receiving regular dialysis thrice a week. His serum creatinine level at admission was 16.1 mg/dL, and he was anuric. The patient had a history of hypertension and anemia, managed with oral antihypertensive medication and regular erythropoietin stimulating agent therapy for renal anemia, respectively. His hemoglobin level at admission was 11.5 g/dL. Other preoperative laboratory tests of the patient indicated the following: WBC 6.44 × 10^3^/μL, platelet count 143 × 10^3^/μL, fasting blood glucose 89.3 mg/dL, prothrombin time 13.0 s, activated partial thromboplastin time 40.1 s, alanine aminotransferase (ALT) 8 U/L, aspartate aminotransferase (AST) 14 U/L, total serum protein 6.5 g/dL, albumin 4.3 g/dL, total bilirubin 0.23 mg/dL, blood urea nitrogen 80.1 mg/dL, serum potassium 5.87 mEq/dL, serum calcium 8.36 mg/dL, and estimated glomerular filtration rate (eGFR) 3.4 ml/min/1.73 m^2^. Serologic tests for his hepatitis B virus (HBV) markers, and quantitative antibody tests for hepatitis C virus (HCV), HIV, and Treponema pallidum (syphilis) were all negative. The patient's pre-transplant blood pressure was 154/82 mmHg, body temperature was 36.3°C, heart rate was 61 beats per minute, and body mass index (BMI) was 23.3 kg/m^2^. The patient did not receive a native kidney biopsy, and the etiology of his end-stage renal disease remained unknown. The patient also had a long-standing history of an untreated left inguinal hernia. He denied any history of coronary artery disease, diabetes mellitus, infectious diseases, drug allergies, blood transfusions, or previous surgeries.

**Figure 1:**
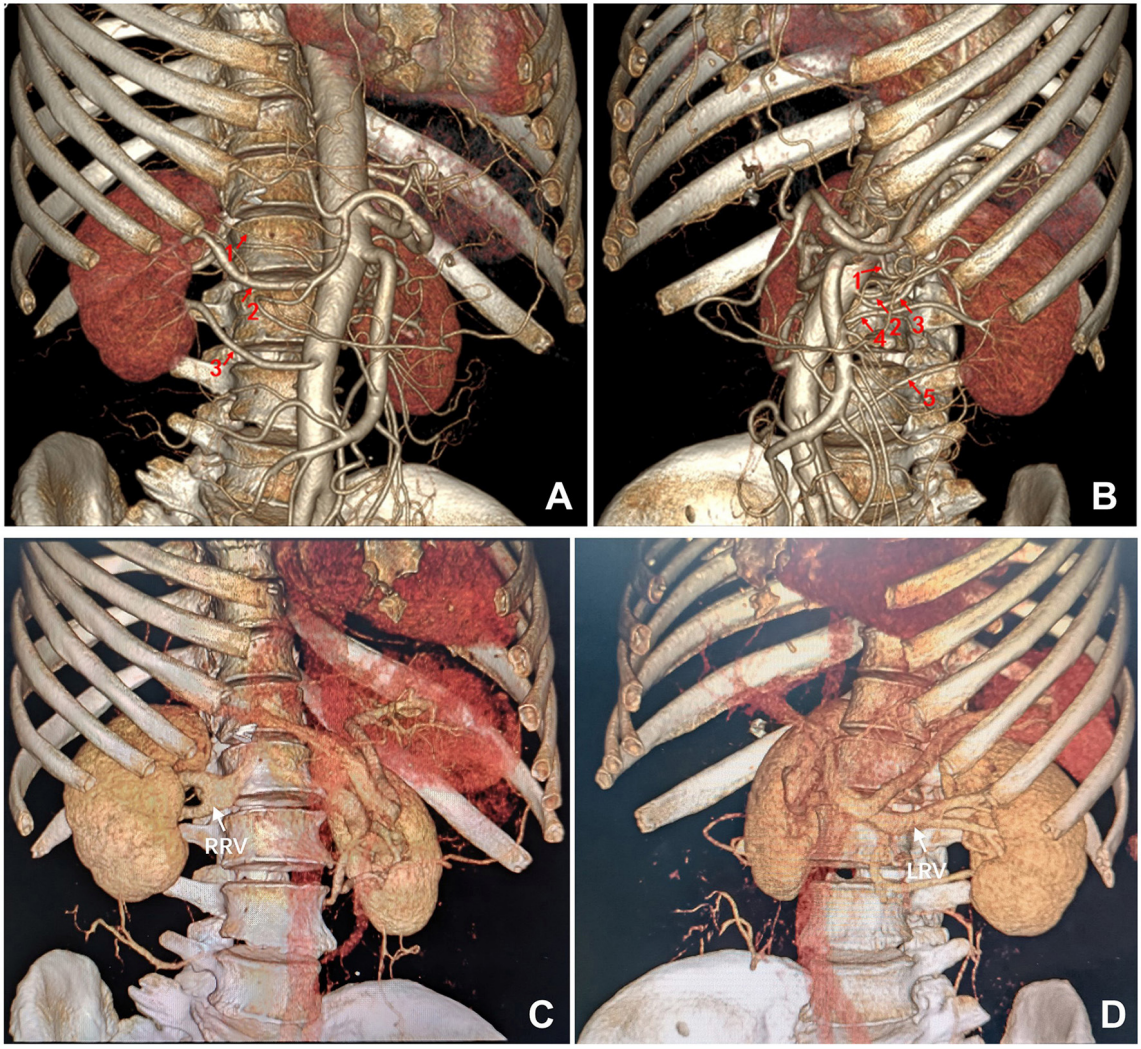
Preoperative computed tomography angiography of the donor. **(A)** Three renal arteries supplying the right kidney. **(B)** Five renal arteries supplying the left kidney. **(C)** Two right renal veins forming a common trunk. **(D)** Two left renal veins forming a common trunk. LRV, left renal vein; RRV, right renal vein.

The donor, the recipient's 65-year-old mother, was diagnosed with hypertension 1 year prior and has since maintained well-controlled blood pressure with a single calcium channel blocker medication. Five years ago, she experienced a history of cerebral hemorrhage from which she recovered fully, without residual symptoms. Single-photon emission computed tomography (ECT) examination revealed a glomerular filtration rate (GFR) of 36.57 ml/min/1.73 m^2^ for her left kidney and 37.22 ml/min/1.73 m^2^ for the right kidney. Her fasting blood glucose and routine urinalysis were within normal limits. Renal artery computed tomography angiography (CTA) demonstrated three arteries supplying her right kidney, including a notably slender superior polar artery (Figure 1A). Her left kidney exhibited five arterial branches, four of which entered through the hilum and one serving as an inferior polar artery, with all five demonstrating relatively uniform caliber (Figure 1B). Both kidneys displayed dual renal veins converging into a common trunk (Figures 1C, D).

Both the donor and recipient shared blood type B, Rh-positive. This marks the recipient's first transplant, and pre-operative panel reactive antibody screening yield negative results. Human leukocyte antigen typing revealed two mismatches across the A, B, and DR 6 loci (Table 1). Pre-transplant complement-dependent cytotoxicity crossmatching between donor and recipient was negative.

**Table 1:** Donor-recipient HLA typing.

**HLA**	**A**	**B**	**DRB1**	**DQB1**
Donor	26:01, 66:01	14:02, 08:01	03:01, 07:01	02:01, 02:01
Recipient	33:01, 66:01	14:02, 58:01	03:01, 07:01	02:01, 02:01

**Figure 2:**
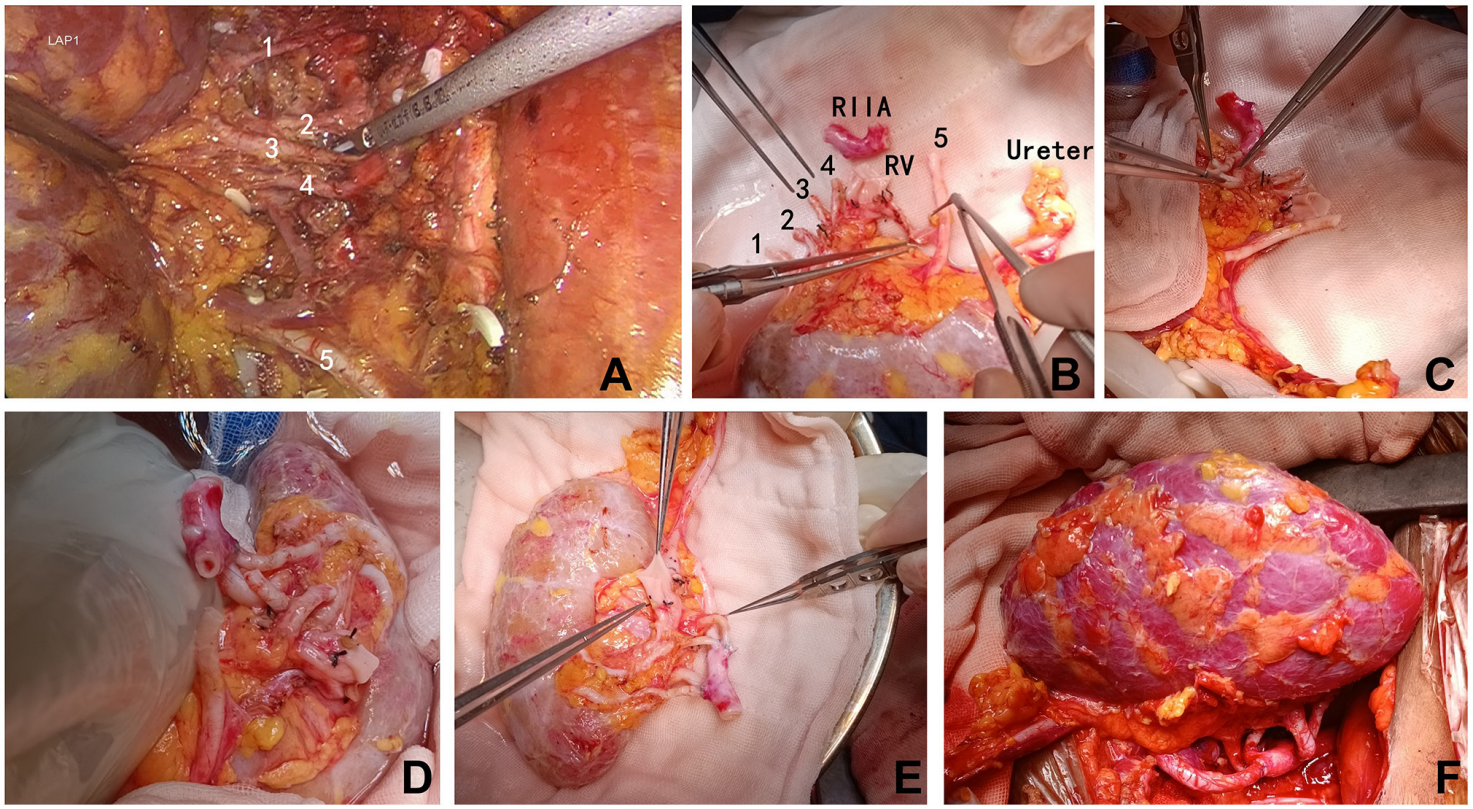
Intraoperative images. **(A)** Retroperitoneoscopic dissection of the five renal arteries supplying the left donor kidney. **(B–E)** Back-table reconstruction of the five donor renal arteries into a single trunk using the recipient's internal iliac artery. **(F)** End-to-side anastomosis of the reconstructed donor renal arterial trunk to the recipient's external iliac artery, and end-to-side anastomosis of the donor renal vein to the recipient's external iliac vein. **(F)** Following reperfusion, the donor kidney exhibited excellent perfusion with a healthy pink hue and no evidence of ischemic areas. RIIA, recipient's internal iliac artery; RV, renal vein.

The donor underwent a left retroperitoneoscopic nephrectomy to procure the left donor kidney (Figure 2A). The procedure was uneventful, with successful dissection of five renal arteries, consisting with pre-operative CTA findings. Warm ischemia time, defined as the interval between clamping the first renal artery with a Hem-o-lok clip and initiating kidney perfusion with histidine-tryptophan-ketoglutarate (HTK) organ perfusion and preservation solution, was 6 min. The total surgical duration was 2.6 h. Immediately following procurement, the donor kidney was submerged in 0–4°C HTK solution and thoroughly perfused to ensure uniform pallor across all renal segments. The recipient, desiring concurrent repair of a long-standing left inguinal hernia, underwent kidney transplantation through a left Gibson incision, incorporating high ligation of the hernia sac. The recipient's left internal iliac artery was meticulously dissected, preserving multiple distal branches. A segment of the internal iliac artery trunk with four distal branches was successfully procured (Figure 2B).

*Ex vivo* vascular reconstruction (procedure lasted ~45 min) was performed on a back table under a 2.5x surgical microscope. The two superior renal arteries (1 & 2, Figure 1B) were first united via a side-to-side anastomosis at their distal ends (Figure 2C). This conjoined segment was then anastomosed end-to-end to the first lateral branch originating from the distal end of the procured internal iliac artery trunk (Figure 2D). The remaining three renal arteries (3, 4 & 5, Figure 1B) were individually anastomosed end-to-end to the other branches of the internal iliac artery, effectively reconstituting the five donor renal arteries into a single arterial ostium (Figure 2E). This reconstructed arterial trunk was then anastomosed end-to-side to the recipient's ipsilateral external iliac artery. The donor renal vein was anastomosed end-to-side to the recipient's external iliac vein. Upon reperfusion, the allograft exhibited excellent perfusion with a healthy pink hue (Figure 2F). Robust pulsations were palpable in all five arterial branches and the renal parenchyma. We did not perform a renal biopsy during surgery.

**Figure 3:**
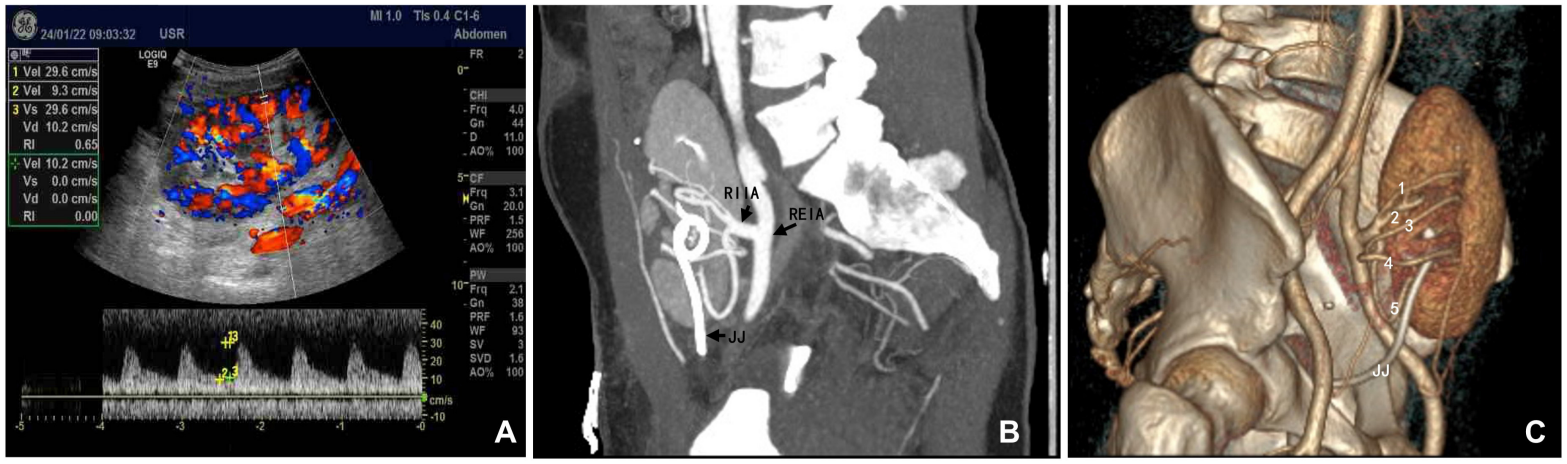
Postoperative evaluation of the transplanted kidney. **(A)** Color Doppler ultrasound performed on the day of surgery demonstrating robust blood flow within the kidney graft. **(B, C)** Computed tomography angiography performed 2 weeks post-transplantation, clearly delineating all five renal arteries. RIIA, recipient internal iliac artery trunk; REIA, recipient external iliac artery; JJ, double-J ureteral stent within the transplanted kidney.

The interval between removal of the transplanted kidney from the 0–4°C HTK perfusion solution and the completion of recipient vessel anastomosis, thereby restoring blood flow, spanned 33 min. Postoperative Doppler ultrasound examinations on the day of surgery and on postoperative days 1, 2, 8, and 9 revealed normal graft architecture, perfusion, and arterial resistance indexes (Figure 3A). Angiography performed on postoperative day 10 confirmed patent anastomoses (Figures 3B, C). The recipient received an induction immunosuppression with basiliximab (Simulect) and methylprednisolone. His maintenance oral immunosuppressive regimen consisted of triple therapy with tacrolimus, mycophenolate, and prednisone. Renal function recovered immediately, obviating the need for dialysis. The recipient's serum creatinine level at discharge on postoperative day 12 was 1.20 mg/dL. Other discharge laboratory findings also revealed good recovery of the patient (Supplementary Table 1). After discharge, the patient underwent our center's regular protocol of follow-up visits: once a week during the first 3 months postoperatively, once every 2 weeks from 3 to 6 months, once a month from 6 to 12 months, and once every 2 to 3 months after 1 year. The frequency of follow-up can be increased if clinically indicated. At 60 days post-transplant, the patient remained in excellent condition, with no evidence of hernia recurrence and a serum creatinine level of 1.27 mg/dL. At 90 days postoperatively, the patient developed BK viruria. After a reduction in mycophenolic acid (MPA) immunosuppressant in outpatient, the urinary BK virus DNA copy number gradually declined and eventually became negative, without affecting the function of the transplanted kidney. No other complications occurred during the perioperative period or the follow-up.

## Discussion

In LLDN, procuring a kidney with MRA generally takes longer than procuring one with a single renal artery (12). It is widely recommended to prioritize the procurement of the left kidney due to its longer renal vein, which makes the procedure technically easier and carries lower surgical risks (13). Nonetheless, laparoscopic procurement of the right kidney or kidneys with MRA is also considered safe and feasible (14), and a recent meta-analysis published in 2023 confirmed single vs. MRA in living donor kidney transplant had similar graft survival and overall survival rates (6). However, there have been no detailed reports on LLDN of kidneys with five renal arteries.

At our center, there is no absolute upper age limit for living donors, but the ECT-based GFR of donor's each single kidney (left or right) must be >35 ml/min/1.73 m^2^. Eligible donors should either be normotensive or have well-controlled hypertension managed with no more than two antihypertensive oral medications at conventional dosages, and total proteinuria level lower than 300 mg/24h. We perform retroperitoneal LLDN for all living donor kidney transplants. When the GFR difference between the donor's left and right kidneys is <10%, we consistently prioritize procuring the left kidney. In this case, the donor's left and right kidneys had comparable GFR values. The right kidney had three renal arteries, but the superior polar artery was extremely thin, complicating vascular reconstruction. Additionally, the right renal vein had a bifurcated trunk, which would result in a short, dual-branch renal vein after LLDN, adding challenges to graft implantation. In contrast, the donor's left kidney, although possessing five renal arteries, had arteries that were relatively uniform in diameter and sufficiently long. Furthermore, while the left renal vein was also bifurcated, it had an adequately long common stem, which was advantageous for vascular reconstruction and graft implantation after LLDN of the left kidney.

There are various techniques for *ex vivo* reconstruction of MRA in living donor kidney transplantation. Options include using deceased donor vessels, the donor's gonadal vein, or the recipient's inferior epigastric artery for reconstruction. Alternatively, MRA from the donor kidney can be merged through end-to-side or side-to-side anastomosis of themselves during *ex vivo* reconstruction (8). Another approach involves separate end-to-side anastomoses of the donor's MRA to the recipient's external iliac artery (Supplementary Figure 1, previously unreported). A well-established strategy is to harvest the recipient's internal iliac artery trunk and its branches for *ex vivo* reconstruction of donor kidney's MRA. This can be done by performing end-to-end anastomoses between the internal iliac artery branches and the donor's renal arteries (15), or by opening the internal iliac artery trunk to create an arterial patch on which the MRA are reconstructed (16). In this case, we successfully applied the former strategy (15), restoring robust blood supply to each renal artery of the kidney. Unlike the reported cases (15), the kidney in this case was procured by LLDN.

In this case, if the surgery had been performed in the right iliac fossa, it would have been technically easier, as the right iliac vessels are more superficial than the left. However, to simultaneously address the recipient's left inguinal hernia, the decision was made to transplant the kidney into the left iliac fossa. This choice demonstrated that using the recipient's left internal iliac artery for *ex vivo* reconstruction of the donor kidney's MRA is feasible, as confirmed by postoperative ultrasound and CTA of the transplanted kidney.

## Conclusion

In summary, although this report was limited by its single-case nature, it suggested that retroperitoneal laparoscopic procurement of a living donor kidney with up to five renal arteries is feasible, and that reconstructing such complex renal vasculature *ex vivo* using the recipient's internal iliac artery is a clinically viable strategy.

## Data Availability

The original contributions presented in the study are included in the article/Supplementary material, further inquiries can be directed to the corresponding author.

## Supplementary material

Supplementary Figure 1 Addressing MRA of the donor kidney through multiple end-to-side anastomoses on the recipient's external iliac artery (REIA). **(A)** A donor kidney has four renal arteries. Three arteries originating from a common trunk (labeled #1) near the upper pole of the kidney are planned to be anastomosed end-to-side to one site on the REIA, while the fourth artery (labeled #2) near the lower pole is designed to be separately anastomosed end-to-side to another site on the REIA. **(B)** The arrows indicate the two separated anastomosis sites (labeled #1 & #2) on the REIA for end-to-side anastomoses. **(C)** Appearance after completing the anastomoses and restoring blood flow. The white arrows indicate the locations of the two separate anastomoses on the REIA, and the black arrow indicates the anastomosis site between the donor renal vein and the recipient's external iliac vein.

Supplementary Table 1 Laboratory test results at the time of patient discharge.
