# Supplementary figures and images for: Case Report: Successful transplantation of a living donor kidney with five renal arteries procured via laparoscopy and back-table vascular reconstruction using the recipient's internal iliac artery
Source: Front Med (Lausanne). 2025 Jul 25;12:1553478. doi: 10.3389/fmed.2025.1553478 (PMC12331733; doi:10.3389/fmed.2025.1553478)

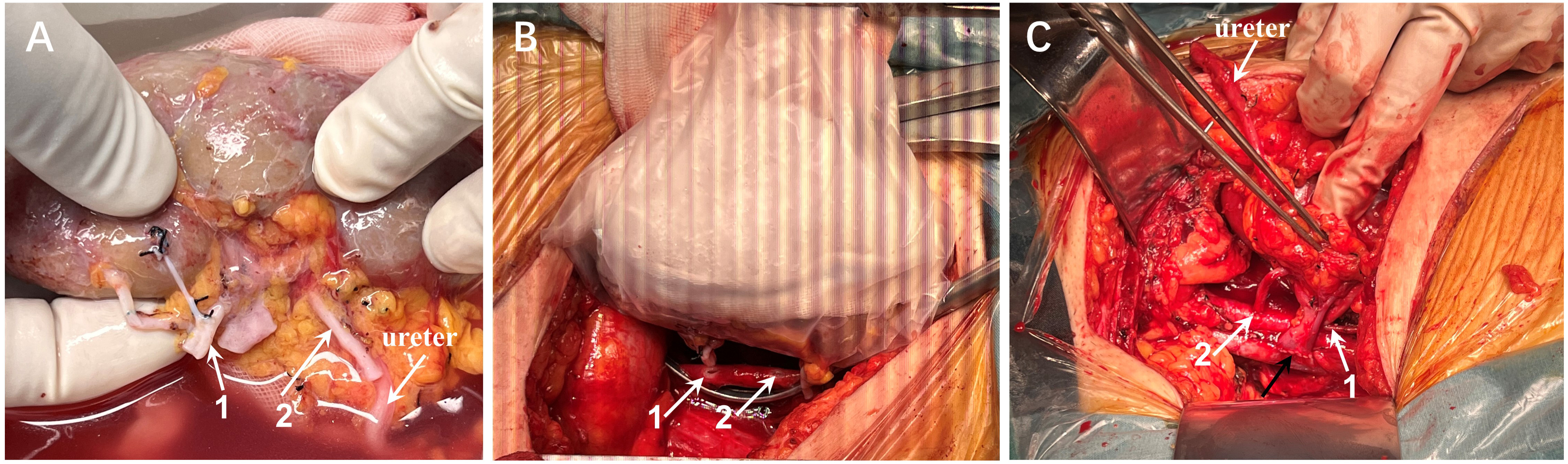

Supplement: Supplementary Figure 1 — Addressing MRA of the donor kidney through multiple end-to-side anastomoses on the recipient's external iliac artery (REIA). (A) A donor kidney has four renal arteries. Three arteries originating from a common trunk (labeled #1) near the upper pole of the kidney are planned to be anastomosed end-to-side to one site on the REIA, while the fourth artery (labeled #2) near the lower pole is designed to be separately anastomosed end-to-side to another site on the REIA. (B) The arrows indicate the two separated anastomosis sites (labeled #1 & #2) on the REIA for end-to-side anastomoses. (C) Appearance after completing the anastomoses and restoring blood flow. The white arrows indicate the locations of the two separate anastomoses on the REIA, and the black arrow indicates the anastomosis site between the donor renal vein and the recipient's external iliac vein. [file Image_1.jpg]
