# Supplementary material for: Case Report: Successful transplantation of a living donor kidney with five renal arteries procured via laparoscopy and back-table vascular reconstruction using the recipient's internal iliac artery
Source: Front Med (Lausanne). 2025 Jul 25;12:1553478. doi: 10.3389/fmed.2025.1553478 (PMC12331733; doi:10.3389/fmed.2025.1553478)
Supplement: Supplementary Table 1 — Laboratory test results at the time of patient discharge. [file Table_1.docx]

Supplementary Table 1. Laboratory test results at the time of patient discharge.

| Laboratory parameters | Laboratory values |
| --- | --- |
| White blood cell count (WBC) | 7.1×10^3^/μL |
| Hemoglobin (Hb) | 9.9 g/dL |
| Platelet count (PLT) | 221.0 ×10^3^/μL |
| Fasting blood glucose | 109.8 mg/dL |
| Alanine aminotransferase (ALT) | 31 U/L |
| aspartate aminotransferase (AST) | 21 U/L |
| Total protein | 5.9 g/dL |
| Albumin | 3.9 g/dL |
| Globulin | 2.1 g/dL |
| Total bilirubin | 0.20 mg/dL |
| Direct bilirubin | <0.12 mg/dL |
| Total cholesterol | 117.2 mg/dL |
| Potassium (K) | 4.5 mEq/L |
| Sodium (Na) | 143.2 mEq/L |
| Calcium (Ca) | 8.8 mg/dL |
| Blood urea nitrogen (BUN) | 23.5 mg/dL |
| Bicarbonate (HCO_2_^-^) | 20.4 mEq/L |
| Estimated glomerular filtration rate (eGFR) | 61.8 ml/min/1.73 m^2^ |
| Tacrolimus trough level | 8.2 ng/ml |
| Blood BK virus DNA | undetectable |
| Urinary BK virus DNA | undetectable |
| Urine protein | 1+ |
